# Chlorhexidine still has skin in the game

**DOI:** 10.1186/s13054-020-03279-6

**Published:** 2020-09-21

**Authors:** Alexandra Lackey, Brandon Kalivoda, Amay Parikh

**Affiliations:** 1 Internal Medicine Division, Department of Internal Medicine, AdventHealth Orlando, 601 E Rollins Street, Orlando, FL 32804 USA; 2 Department of Critical Care, AdventHealth Orlando, 2501 N Orange Ave, Suite 401, Orlando, FL 32804 USA

To the editor:

We read with great interest the recent study by Buetti et al. that described similar infection risk of short-term central venous or arterial catheters covered with either chlorhexidine (CHG) gel or sponge dressings [1]. It was additionally concluded that concomitant use of CHG for skin antisepsis may significantly increase contact dermatitis. In this investigation, contact dermatitis was subjectively reported by nurses when there were any findings beyond “normal skin” including “mild redness only” during dressing changes and line removal. This methodology is simple, but it requires adequate training for the results to be correctly interpreted and used [2].

Despite this, we still favor CHG over povidone-iodine antisepsis, as multiple trials collectively reveal significant reduction in catheter-related blood stream infections by up to 50% [3]. Although severe contact dermatitis may theoretically increase the risk of major catheter-related infection due to skin breakdown, concern for this complication should not preclude use of CHG dressings. In a case series of seven patients with erosive irritant contact dermatitis due to CHG-containing dressings, simply switching to an alternative antimicrobial dressing led to resolution of the lesions. Most notably, extensive infectious workup for the included patients was negative [4]. Alternative options for CHG-sensitive individuals may include topical antibiotics, silicone and silver-impregnated dressings, and cleansing with alcohol and povidone-iodine.

Many potential materials for central lines and their dressings remain unstudied. Although chlorhexidine and silver sulfadiazine-impregnated catheters have been investigated, these interventions have not been found to significantly reduce catheter-related bloodstream infections compared to standard catheters [5]. However, trials seeking statistically significant conclusions regarding major catheter-related infections must compose a very large sample size due to the small number of events that occur with the current standard of care.

With this in mind, we thoroughly appreciate the high-quality analysis of 3700 catheters with CHG-impregnated dressings set forth by Buetti and colleagues.

## Data Availability

Data sharing not applicable to this article as no datasets were generated or analyzed during the current study.
