# Supplementary material for: Chlorhexidine still has skin in the game
Source: Crit Care. 2020 Sep 21;24:563. doi: 10.1186/s13054-020-03279-6 (PMC7504838; doi:10.1186/s13054-020-03279-6)
Supplement: Related texts

# Reply: Authors’ response: Alcoholic 2% chlorhexidine for skin disinfection should be used

**Authors:** Niccolò Buetti, Jean-François Timsit

**Affiliations:** 3 grid.10988.38 0000 0001 2173 743X University of Paris, INSERM IAME, U1137, Team DesCID, Paris, France; 4 grid.411119.d 0000 0000 8588 831X Medical and Infectious Diseases Intensive Care Unit, AP-HP, Bichat-Claude Bernard University Hospital, 46 rue Henri Huchard, 75877 Paris Cedex, France; 5 grid.150338.c 0000 0001 0721 9812 Infection Control Programme, University of Geneva Hospitals and Faculty of Medicine, Geneva, Switzerland

To the editor,

We read with great interest the letter by Lackey et al. and their statement on chlorhexidine gluconate (CHG) skin disinfection and CHG-impregnated dressings [6].

The aim of our study was to perform a comparison between CHG-impregnated sponge-dressing and CHG-gel-dressing [1]. A supplementary analysis showed that concomitant use of CHG for skin antisepsis and CHG-impregnated dressing may increase contact dermatitis among CHG-gel-dressings. We agree with Lackey et al. that the outcome ICDRC ≥ 1 should interpreted with caution. Therefore, we performed a sensitivity analysis using as outcome an ICDRC ≥ 2 (i.e., “red and slightly thickened skin”). This sensitivity analysis confirmed the results of the main analysis using as outcome an ICDRC ≥ 1 [1]. Moreover, we performed a supplementary analysis for addressing this question using the *ordinal* variable “ICDRC”: we used mixed effects ordinal logistic regression models for clustered data (PROC GENMOD of SAS) with the response variable ICDRC, and we adjusted for the time between catheter insertion and dressing change and the ICU. The odds ratio for the increase by one ICDRC degree at each dressing change was 4.71 (95% CI 2.12–10.46, *p* < 0.001) for Gel-dress (versus Sponge-dress). Therefore, our results were confirmed taking into account all ICDRC degrees of contact dermatitis.

We completely agree with Lackey et al. regarding CHG skin disinfection. The most recent meta-analysis on this topic showed that CHG skin disinfection reduced catheter-related bloodstream infections (CRBSI) although the evidence was low grade [7]. These findings were confirmed by the CLEAN trial that illustrated the superiority of alcoholic 2% CHG (versus alcoholic 5% povidone iodine) in CRBSI prevention [8]. We are, therefore, convinced that alcoholic 2% CHG should be used for skin antisepsis prior to any intravascular catheter insertion in the ICU [9]. However, the additional benefit in CRBSI prevention of CHG-impregnated dressings after skin antisepsis with alcoholic CHG 2% remains unknown [9]. In our opinion, the use of CHG-impregnated dressings should be considered only in ICUs having infection rates above the institutional goals [9]. Moreover, in our analysis, only alcoholic ≤ 0.5% CHG was used: the frequency and impact of contact dermatitis using concomitantly 2% CHG for skin disinfection and CHG-impregnated dressings remain an unresolved issue.
